# Results of a Systematic Literature Review on AI in Aging: Evidence and Insights

**DOI:** 10.1093/geroni/igaf122.2256

**Published:** 2025-12-31

**Authors:** Kimia Tuz Zaman, Wordh Ul Hasan, Juan Li, Bo Xie

**Affiliations:** North Dakota State University, Fargo, North Dakota, United States; North Dakota State University, Fargo, North Dakota, United States; North Dakota State University, Fargo, North Dakota, United States; The University of Texas at Austin, Austin, Texas, United States

## Abstract

The rapidly aging population necessitates innovative healthcare solutions. This review synthesizes current evidence on artificial intelligence (AI) applications in older adult care to inform stakeholders about clinical, functional, and psychosocial outcomes. (Details of our AI-assisted systematic literature review method are reported elsewhere; here we focus solely on the findings.) Following PRISMA 2020, an initial PubMed search yielded 857 records, with 57 meeting inclusion criteria after automated filtering and manual validation. Data were extracted using AI agents addressing 12 predetermined research questions on AI technologies, outcome measures, stakeholder perspectives, and implementation challenges. Most studies employed machine learning and natural language processing in home-based and telehealth settings. Thematic analysis identified five key domains: (1) health monitoring and prediction, where fall risk models and chronic disease predictors achieved accuracy rates above 70%; (2) social and emotional support, with AI-driven voice assistants and reminiscence therapy significantly reducing loneliness; (3) cognitive assessment, as sensor-based gait analysis demonstrated high sensitivity (0.961) and AI-driven music therapy improved cognitive scores (p < .05); (4) variable implementation effectiveness across studies; and (5) challenges including technical infrastructure, data privacy, and regulatory issues. Notably, several studies reported improvements in balance outcomes, while others documented moderate accuracy in detecting atrial fibrillation and diabetes, underscoring the diversity of AI applications. Cost-effectiveness, privacy concerns, and integration with care systems were recurrent themes. These findings underscore the potential of AI to enhance older adult care, although short follow-up periods limit long-term conclusions. Future research should address these barriers to facilitate broader AI integration in healthcare.

